# The lived experiences of frontline nurses during the coronavirus disease 2019 (COVID‐19) pandemic in Qatar: A qualitative study

**DOI:** 10.1002/nop2.901

**Published:** 2021-05-05

**Authors:** Ralph C. Villar, Abdulqadir J. Nashwan, Rejo G. Mathew, Ahmed S. Mohamed, Sathish Munirathinam, Ahmad A. Abujaber, Mahmood M. Al‐Jabry, Mujahed Shraim

**Affiliations:** ^1^ Department of Nursing Hazm Mebaireek General Hospital Hamad Medical Corporation Doha Qatar; ^2^ University of Calgary in Qatar Doha Qatar; ^3^ Department of Public Health College of Health Sciences QU Health Qatar University Doha Qatar

## Abstract

**Aim:**

This study aims to explore the lived experiences of frontline nurses providing nursing care for COVID‐19 patients in Qatar.

**Design:**

Qualitative, Phenomenological.

**Methods:**

Nurses were recruited from a designated COVID‐19 facility using purposive and snowball sampling. The participants were interviewed face‐to‐face using semi‐structured interview questions from 6 September–10 October 2020. The interviews were transcribed and analyzed using Colaizzi's phenomenological method.

**Result:**

A total of 30 nurses were interviewed; (76.7%) were deployed for >6 months. Three major themes were drawn from the analysis: (a) Challenges of working in a COVID‐19 facility (subthemes: working in a new context and new working environment, worn out by the workload, the struggle of wearing protective gear, fear of COVID‐19, witnessing suffering); (b) Surviving COVID‐19 (subthemes: keeping it safe with extra measures, change in eating habits, teamwork and camaraderie, social support); and (c) Resilience of Nurses (subthemes: a true calling, a sense of purpose).

## 1 INTRODUCTION

The Coronavirus Disease 2019 (COVID‐19) has incredibly overwhelmed hospitals and health‐care workers since it was first discovered in December 2019 in Wuhan, China with 62,363,527 confirmed cases and 1.46 million recorded deaths worldwide as of December 1, 2020 (Hui et al., 2020). The SARS‐CoV‐2 (Severe Acute Respiratory Syndrome Coronavirus 2) had found its way to Qatar on the 29 February 2020 when a patient was evacuated from the Islamic Republic of Iran. Based on the latest report of the Ministry of Public Health (MoPH) in Qatar, the total number of cases in the country was 1,112,430 (Al Kuwari et al., 2020; Iqbal et al., 2020; Nair et al., 2020; Soliman et al., 2020). One of the significant key players were the nurses who were maximized to perform multitasks over extended hours to provide nursing care for COVID‐19 patients.

The sharp surge in COVID‐19 cases is associated with significant increase in nurse staffing demands as health‐care authorities all over the world had to immediately deploy and train nurses to work in the medical/surgical, emergency and critical care units (Minissian et al., 2020). This can cause an enormous amount of stress, fear and lack of confidence to health‐care providers because they have to treat a disease which was not well understood and out of their line of expertise; furthermore, they had to learn and master new technical skills in a short time (Liu, Luo, et al., 2020). In Qatar, the health authorities have deployed some nurses working in governmental hospitals to COVID‐19 designated facilities and the recruitment of nurses (who are currently in the country with valid nursing license and willing to work in a COVID‐19 facility) was accelerated. Orientation and training were fast tracked to hasten the deployment of nurses to different COVID‐19 facilities in Qatar. For instance, existing government employee nurses who worked in post anaesthesia care units were given at least 10 days to train under a preceptor to work in critical care units.

Because of the change in the working environment, health‐care providers had to work in areas that did not meet infection control standards. They were confused about the difference in protocols like prescribing and carrying out doctor's orders (Liu, Luo, et al., 2020). In addition, different studies have shown that health‐care professionals who were treating COVID‐19 patients have higher risks of physical and mental health problems due to insomnia, stress, anxiety and depression (Liu, Luo, et al., 2020; Lu et al., 2020; Xiao et al., 2020).

According to the International Council of Nurses (ICN) report, around 2,262 deaths in nurses has been listed and more than 1.6 million got infected. In addition to ICN's report there is an alarming number of burnout and exhaustion among nurses leading them to quit their jobs (ICN, 2021). The safety and health of the frontline nurses are critical to ensure safe and quality nursing care to patients and is vital in the quest to overcome the COVID‐19 crisis or future pandemics. Different studies had shown that nursing is a profession that accumulates tons of stress that has an alarming effect on the physical, mental and social well‐being especially during outbreaks (Janda & Jandová, 2015; Lee et al., 2018; Liu, Luo, et al., 2020; Lu et al., 2020; Marjanovic et al., 2007; Torales et al., 2020). Nurses in Qatar are working tirelessly since the beginning of the pandemic. However, little is known about the experiences and effect of the COVID‐19 pandemic on frontline nurses in Qatar. Therefore, the aim of this study was to assess the experience of frontline nurses providing nursing care for COVID‐19 patients in Qatar.

## 2 METHODS

To uncover the meaning of the frontline nurses' experience during the COVID‐19 pandemic, a qualitative, phenomenological research design was used. Phenomenology explores how individuals make sense of the world in terms of meanings they create (Pope & Mays, 2020). The purpose of phenomenological research is to focus on the lived experiences of the participants. It emphasizes the importance of personal perspective and interpretation.

A semi‐structured interview guide question was used in this study to collect data. Probing questions were formulated to get an in‐depth understanding of the participants' experiences guided by the participants' initial responses. The participants were selected through purposive and snowball sampling. Interviews were conducted face to face while observing infection control precautions in a private room within the study site (RCV, RGM, ASM and SM conducted the interviews). Four open‐ended questions were first asked: (a) Can you describe to me your experience of taking care of patients with COVID‐19? (b) What were the differences in working in a COVID‐19 facility from your previous working environment? (c) What were your feelings, thought processes or emotions when you were providing nurse care for COVID‐19 patients? and (d) How did you cope up with the COVID‐19 crisis? Probing and follow‐up questions were then formulated to get an in‐depth understanding of the participants' experiences guided by the participants' initial responses, examples were: “Can you specify the difficulties you mentioned in providing care to patients with COVID‐19”; “Based on your experience during this pandemic, what are your nursing career plans 1–3 years from now”; “What else could you have done more”; “How did you feel?”; “Can you tell me more or can you expand on this please”, and so on. With the participant's permission, interviews were audio‐recorded to aid in the accurate transcription and analyzed using Colaizzi's phenomenological method. All participants provided written informed consent.

### 2.1 Study population and location

The study was conducted in Hazm Mebaireek General Hospital (HMGH), a designated COVID‐19 facility in Qatar, which extended its capacity to 560 beds to provide quality care to most patients with moderate to severe symptoms of COVID‐19.

The participants were enrolled in the study using purposive and snowball sampling. The first 10 participants were recruited from their departments as identified by the researchers to be eligible, considering that they were: (a) registered nurses who took care of patients with COVID‐19 in Qatar; (b) registered nurses who were assigned to HMGH for at least one month during the COVID‐19 pandemic; and (c) were willing to participate in the study. The other participants were recruited through snowball sampling.

### 2.2 Study procedure

Interviews were done face to face while observing infection control protocols and the conversations were audio‐recorded to facilitate transcriptions of the responses which was done 24 hr from the interview and was reviewed by two researchers of this study. Identifiers were removed from the transcripts and codes were used to label participants (e.g., N1, N2, N3, etc.).

Interviews were done from 6 September–10 October 2020. The participant's characteristics were obtained before the interview. The participants were informed that they have the right to withdraw from the study at any time should they decide not to participate in further sessions. The schedule of the interview was based on the participant's availability and convenience. The data, including the audio‐recorded files, was stored in the protected computer by Hamad Medical Corporation (HMC) in accordance with the corporate policies and guidelines.

### 2.3 Analysis

**FIGURE 1:**
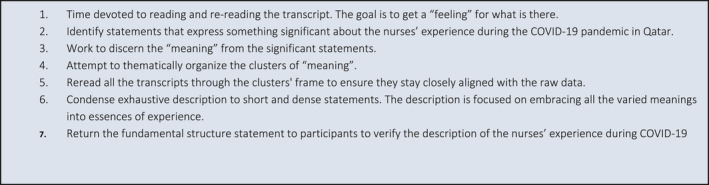
Collaizi's method of data analysis

In this study, Collaizzi's seven‐step procedure analysis for the phenomenological method was followed [58]. The data and themes were developed by RCV and RGM and discussed by all authors. Colaizzi's seven‐step method of analysis is seen in Figure 1.

### 2.4 Ethics approval

The study was approved by the Medical Research Center (MRC) – Institutional Review Board (IRB) at Hamad Medical Corporation (MRC‐01‐20‐423).

## 3 RESULTS

Our sample consisted of 30 nurses as seen in Table 1. The mean age of the participants was 31 years (standard deviation (*SD*) 2.8 years), with a mean work experience of 9.1 years (*SD* 2.7 years) and with a mean work experience in Qatar of 2.5 years (*SD* 0.9 years). 66.7% of participants were married. Four participants (13.3%) were women. The participants were from different specialties, with a total of nine participants from Emergency Care, six from Medical‐Surgical units, six from the Critical Care Unit, five from Ambulatory Care Unit, and four from the Perioperative Department. Eleven participants (30%) were deployed to units different from the units that they worked in before the COVID‐19 crisis. Among them, five nurses were deployed from outpatients to inpatient wards and emergency rooms, four perioperative nurses were deployed to critical care units and emergency rooms, one critical care nurse was deployed to an inpatient ward, and one school nurse (with neonatal, medical‐surgical background) was deployed to an inpatient ward. The remaining 19 participants were assigned to the same units where they worked previously. None of the participants experienced working in a large‐scale pandemic like the current COVID‐19 pandemic. Amongst the participants, 76.7% were deployed for 6 months or more, 10% were deployed for 4–5 months, and the remaining were deployed for a month.

**TABLE 1:** Characteristics of participants

Code	Age	Gender	Marital status	Years of exp	Years of exp in Qatar	Expertise	Unit deployment	Role	Length of deployment
N1	33	M	Married	11	2.5	Critical care	Critical care	Bedside nurse, charge nurse	≥6 months
N2	37	F	Married	12	10	Critical care	Critical care	Bedside nurse	≥6 months
N3	31	M	Married	10	3	Emergency care	Emergency department	Bedside nurse	≥6 months
N4	31	M	Single	7	3	Critical care	Critical care	Bedside nurse	≥6 months
N5	34	F	Married	12	1.5	Critical care	Critical care	Bedside nurse	≥6 months
N6	27	M	Single	2	2	Emergency care	Emergency department	Bedside nurse	>6 months
N7	35	M	Married	9	3	Perioperative	Inpatient and critical care	Bedside nurse	>6 months
N8	30	M	Single	11	3	Emergency care	Emergency	Bedside nurse	>6 months
N9	33	M	Married	8	3	Emergency care	Emergency department	Bedside nurse	>6 months
N10	28	M	Single	6	2	Critical care	Critical care	Bedside nurse	>6 months
N11	33	M	Married	10	3	Emergency care	Emergency department	Bedside nurse	>6 months
N12	35	M	Married	12	3	Emergency care	Emergency department	Bedside nurse	>6 months
N13	37	M	Married	10	3	Medical/surgical and emergency care	Emergency department	Bedside nurse	>6 months
N14	35	M	Married	8	3	Medical/surgical and emergency care	Emergency department	Bedside nurse	>6 months
N15	25	F	Single	3	5 MONTHS	Critical care	Inpatient	Bedside nurse	4–5 months
N16	34	M	Married	8	2	Emergency care	Emergency department	Bedside nurse	≥6 months
N17	28	M	Single	6	3	Emergency care	Emergency	Bedside nurse	≥6 months
N18	32	M	Single	10	2	Emergency and critical care	Emergency	Bedside nurse	≥6 months
N19	27	F	Married	5 ½	1.5	Medical/surgical, neonatal, school nursing	Inpatient	Bedside nurse	1 month
N20	30	M	Married	8	1.5	Medical surgical	Inpatient	Bedside nurse	≥6 months
N21	31	M	Married	10	1	Medical surgical	Inpatient	Bedside nurse	≥6 months
N22	33	M	Married	11	7	Medical surgical	Inpatient	Charge nurse	≥6 months
N23	32	M	Married	10	3	Outpatient/ambulatory care	Inpatient	Bedside nurse	≥6 months
N24	29	M	Married	6	3	Perioperative	Inpatient/critical care	Bedside nurse	4–5 months
N25	33	M	Married	11	3	Outpatient/ambulatory care	Inpatient/emergency department	Bedside nurse	≥6 months
N26	49	M	Married	26	9	Perioperative	Critical care	Charge nurse	≥6 months
N27	33	M	Single	10	3	Perioperative	Emergency department	Bedside nurse	4–5 months
N28	33	M	Single	12	2	Outpatient/ambulatory care	Inpatient	Bedside nurse	>6 months
N29	33	M	Married	8	3	Outpatient/ambulatory care	Inpatient	Bedside nurse	>6 months
N30	33	M	Single	8	3	Outpatient/ambulatory care	Inpatient/emergency department	Bedside nurse	≥6 months

**FIGURE 2:**
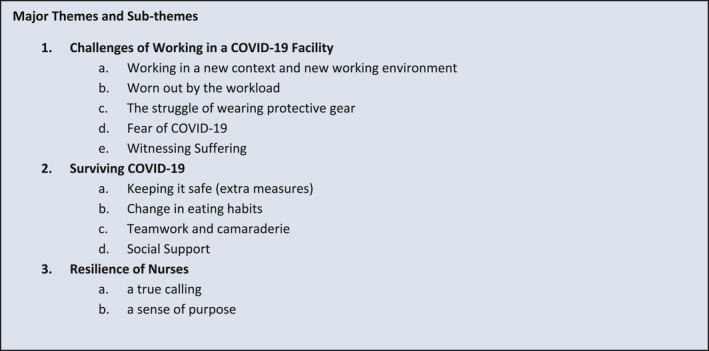
Major themes and sub‐themes

Three major themes were drawn from the analysis as seen in Figure 2.

### 3.1 Challenges of working in a COVID‐19 facility

Nurses in a COVID‐19 facility encountered numerous challenges that impacted their physical, psychological and emotional well‐being. This theme consisted of five sub‐themes relating to the stress from working in a new context and environment workload, wearing PPE's, fear and witnessing patient suffering.

#### 3.1.1 Working in a new context and a new working environment

None of the nurses in this study have worked in a pandemic environment like the current COVID‐19 pandemic. Nurses had to adapt to new policies and pathways. Most of the nurses explained that the sudden change of roles and responsibilities brought much stress, plus the fact that the virus was not yet well understood:

This is a new disease, and we don't know much about it. We had to take extra precautions, understand new medications, and learn new protocols. This is a new experience for us. (N2)

Because of the increasing number of COVID‐19 patients, nurses were deployed and assigned to different units outside of their nursing experience and background. Nurses had to report to a new supervisor, work with new colleagues; be oriented and learn new skills in a short amount of time:

The first three weeks I was assigned to test patients for COVID‐19. When the cases increased, I was deployed to the emergency room to care for acute patients. After some time, I was transferred to the inpatient ward. When you are a new staff you will be trained for at least 3‐months. But during the pandemic, I was immediately assigned to a new role. I didn't know a lot about the routine in the In‐patient and on my first day I received 5 patients plus admissions and all of them needs critical care. Not to mention I had to activate 3‐4 codes in a shift. It was a struggle. (N25)

#### 3.1.2 Worn out by the workload

In the initial stages of the pandemic, the frontline nurses in this study were exhausted due to the surge of patients. Nurses had to take care of more patients than they used to and adjust to demanding shift schedules to support staffing needs. They had more tasks to accomplish and they had to work for extended hours caring for critically ill patients. Most of the nurses felt tired because of the workload:

There was an influx of patients. The first two months were difficult. I started having anxiety attacks and sleepless nights. I always thought about work. I recalled everything that happened throughout the day, whether I did everything right, gave the right medications, or missed anything. (N30)

#### 3.1.3 The struggle of wearing protective gear

Frontline nurses consistently expressed the difficulty of working in full personal protective equipment (PPE). According to the nurses in the study, wearing PPE was very uncomfortable. Because of the airtight N95 masks and the thick gown, nurses were getting suffocated and sweating under the PPEs. Some of the nurses were complaining of headaches and pressure injuries to the face due to prolonged use. Furthermore, wearing full PPEs was a barrier to nutrition and elimination, which is a basic human need. Some of the participants mentioned the limited availability and inconsistent quality of the PPEs:

Wearing PPE was a struggle, you had to wear and remove it in a certain way. It was hard to breathe, move, and communicate. It was also hot underneath all those layers. It was a hassle to remove the PPEs for a drink. I remembered a time I had to keep my N95 for 3 days. It was for a short while until the supplies were replenished. It was suffocating, but you get used to it. (N3)

#### 3.1.4 The fear of COVID‐19

Because of the highly contagious nature of COVID‐19 and the lack of sound information, the participants experienced fear of contracting COVID‐19. Most of the participants were afraid of spreading the virus to their families:

I was afraid of contracting the virus. We don't know a lot about it besides the fact that it was a highly contagious virus and people were dying. I have asthma and I know that I will suffer a lot when it gets to me. Worst, I might even be intubated and admitted to intensive care. Both of my parents were in their senior years. I fear for them. I fear that I might bring the virus to them. Unfortunately, I didn't have anywhere else to stay so I had to isolate myself at home. (N15)

#### 3.1.5 Witnessing suffering

Due to strict isolation measures, relatives were prohibited from visiting their dear ones in the hospital. Nurses felt sad that their patients had to be alone in their most challenging times, some even at the end of their life. Participants felt powerless when their patients deteriorated despite their best efforts and expressed their emotional turmoil in observing their patients suffer from COVID‐19.

I had three patients who passed away from COVID‐19. We did everything we can. I felt sad for the patients. They don't have any clue what's happening to them because they were sedated. They were powerless. At some point, we tried to wake them up and wean them from the ventilators, but we end up intubating again. I felt sad for their family as well. During the pandemic, visitors were restricted. They were not able to see them or provide them support. There were times that I felt powerless too because I wanted to help the patients, but I can't. (N7)

### 3.2 Surviving COVID‐19

Front line nurses during the pandemic faced numerous challenges, physically, psychologically and emotionally brought by COVID‐19. This theme describes the different coping mechanisms or adaption that the nurses in this study used to overcome the challenges of working in a designated COVID‐19 facility.

#### 3.2.1 Keeping it safe (extra measures)

Nurses feared acquiring COVID‐19 and consequently spreading it to their families. Due to their frequent exposure to patients with COVID‐19, nurses felt unsafe despite the use of PPEs and compliance with infection control practices. Because of this, nurses felt the need to take additional measures to protect themselves and their families which they have never done in normal circumstances. These measures came from the nurse's own intuition and were not from any infection control policies or any governing body's recommendations. To address their fear of contamination, nurses took frequent showers and wore additional PPEs on top of their masks, gown and gloves. Some participants also became peculiar in cleaning any items that they might have brought from the hospital as well as their cars.

Sometimes, I wear double gloves and I place a surgical mask on top of my N95 mask. When I get in the car, I rubbed my hands with alcohol and clean the steering wheel with alcohol. Before entering the house, I take off my shoes and ask somebody to open the door and then I go straight ahead to the bathroom. (N26)

Some of the participants were also very particular about maintaining their uniforms at home. Nurses in this study perceived their uniform as a potential risk of spreading the virus at home:

We started using disposable plates and I had to wear a “special uniform” (a new set of scrubs and shoes for COVID use only). After duty, I will call home before stepping into the house and isolate the children in one room while I clean myself up. (N2)

Nurses in this study considered themselves as carriers of the virus. Frontline nurses took what they seem necessary to lessen the risk of transmission by wearing masks at home, avoiding their families, or by isolating themselves in their own homes. Some of the nurses opted to stay in hotels as temporary accommodations:

I limited my interaction with my kids and spent most of my day‐offs inside my car. I only go inside the house to take a shower, eat, and sleep. This had a huge emotional toll on my children and me. (N21)

Some of the participants in this study admitted minimizing their exposure by scheduling their care and limiting the time they spend with patients:

I was afraid of acquiring the virus. I was not able to maximize my care to my patients because I tried not to expose myself a lot. I could have done better. Given the chance I will go back in time and provide the best care I can give. (N24)

#### 3.2.2 Change in eating habits

This theme describes the change in eating habits of nurses in response to COVID‐19. Some of the nurses in this study tend to eat more in response to stress while others believed that eating a lot would increase their immunity to the virus:

I ate a lot during the crisis. I think I gained around 10 kg. I'm exhausted after work; I eat a lot so I can bulk up more energy. I took vitamins more consistently too. I do exercise as well. I limited my alcohol intake during the crisis. I didn't want my immune system to drop. (N27)

In some situations, the nurses mentioned their conscious effort in their food preferences to boost their immunity and maintain a healthy body.

I wasn't a healthy eater, but during the COVID‐19 pandemic I was able to maintain a good diet and an exercise routine. I exercise and walk daily around the neighborhood. Because of that, I was able to bring my cholesterol level down. (N17)

#### 3.2.3 Teamwork and camaraderie

According to the participants in this study, nurses in a COVID‐19 facility in Qatar were able to get the job done because they worked together as a team. According to the participants of the study a good relationship was developed during the most difficult hours even if they had only worked with the other nurses for less than six months.

The support was good; no complaints at all. Every now and then, we talked to each other. We were a family here. You can openly speak or voice out to other charge nurses. I am lucky, I became friends with most of the charge nurses. We were a team. We developed a good relationship. We always looked forward to working together. We developed a camaraderie even though we only met during the COVID‐19 crisis. (N26)

#### 3.2.4 Social support

Nurses in this study sought social support during their battle with COVID‐19. In a new world where social meetings were prohibited, nurses in a COVID‐19 facility found support from their families and people they worked with.

Talking to someone was the best stress reliever. There was no option to visit your friends or relatives due to this pandemic. Even though it's hard to sit alone in your room after the duty, it was the right thing to do. As much as possible, I will call my wife who's in India to talk about the stress at work. Although sometimes you cannot share everything as it might do more harm than good. So, I call some of my colleagues to whom I am really close to and share my troubles. (N22)

### 3.3 Resilience of nurses

Nurses in the study showed resilience to work in the pandemic despite the risks involved. Nurses perceived their role as of utmost importance during this pandemic. They found value and purpose in a time of need and they considered it as a part of their job. Frontline nurses in this study did not find COVID‐19 as a reason to quit but as a driving force to pursue it.

#### 3.3.1 A true calling

Most of the participants in the study described nursing as a vocation. Caring for patients in the time of crisis allowed them to save lives and bring comfort to those in need.

I believe as a nurse it is our job to take care of them. I never thought of quitting. Being a nurse is great because it gives you the capability to help people, regardless of who they are and where they come from. (N27)

#### 3.3.2 A sense of purpose

Participants felt good about what they have accomplished during the COVID‐19 pandemic. Most of the participants mentioned how rewarding it was to see patients recover from the virus and how they felt a strong sense of purpose to care for them.

I never had the idea to resign. It is like giving up on the people who need you and not your career. I feel like I am on a mission. Some of my friends kept on complaining because of the restrictions but I go to work to face the patients every single day and I chose to do this. (N7)

On the other hand, there were instances where we felt like heroes when patients survived and improved. I remember one of my patients who went critical. After 2 weeks, he came back to us. He was better. Experiences like this make you feel proud of what you do. (N20)

## 4 DISCUSSION

This study assessed the experiences of frontline nurses providing nursing care for COVID‐19 patients in Qatar. We have identified three main themes featuring nurses' experiences: challenges related to working in a COVID‐19 facility, surviving COVID‐19 and resilience of nurses. Overall, the results of this study revealed that nurses providing nursing care for COVID‐19 cases in Qatar were able to cope with numerous challenges and showed resilience and willingness to work amidst the pandemic.

Adversities that the nurses encountered most especially during the initial and peak phase of the pandemic were very common. Nurses working in a pandemic like COVID‐19 were more likely to report stress compared to those who were not dealing with patients infected by the virus (Alshekaili et al., 2020; Mo et al., 2020). Our findings are comparable with Liu, Luo, et al., (2020)) and Shoja et al. (2020). Nurses in our COVID‐19 facility were stressed out because they had to face a virus that was not completely understood, they had to adjust to new pathways and policies, master new nursing skills and report to a new unit with less or zero time for training and orientation. During the initial stages of the pandemic, there was insufficient and contrasting information on the treatment, mode of transmission and measures to contain the virus. In the study by Park et al. (2020), reading or hearing about the severity and high contagiousness of COVID‐19 was identified as the most common stressor. None of the nurses in our study have worked in a pandemic with the kind of magnitude of COVID‐19 pandemic. Without proper channel or dissemination of facts, nurses may be exposed to misleading information which can cause higher levels of stress and confusion (Park et al., 2020; Tasnim et al., 2020). Proper channels of information, usage of company‐based information technology and transparency should be promoted to remove misleading information. Nurses should be encouraged to rely on authorized governing bodies for information on COVID‐19 and be discerning on information obtained from social media. Nurses should be briefed daily regarding updates to management of COVID‐19 and related protocols.

The surge of patients during the pandemic caused great challenges to the health‐care workforce. Because of the increasing number of COVID‐19 patients, nurses were deployed and assigned to different units outside of their nursing experience and background. Therefore, they had to adapt to new policies, report to new supervisors, work with new colleagues, be oriented with the physical layout, and learn new procedures. Nurses had to adjust to this new situation in a short period of time. The sudden change of roles and responsibilities brought a lot of stress to the nurses. In Liu, Luo, et al., (2020)), adapting to new working environments and inexperience was linked with feelings of depression and anxiety among health‐care providers. Nurses in Qatar were deployed to ICUs, emergency rooms, and in‐patients with few to no clinical experience in providing nursing care to patients with infectious disease. Although efforts were made to prepare nurses during the pandemic, continuous education and training should be provided to assure that nurses are confident to take care of patients during a pandemic (Liu, Luo, et al., 2020).

Nurses have a high probability of experiencing low professional quality of life because of stress and burnout even prior to COVID‐19 (Kim et al., 2014). Without a doubt, nurses who were caring for COVID‐19 patients have experienced high stress levels and increased risk of mental health conditions (Liu, Luo, et al., 2020; Liu, Yang, et al., 2020; Nashwan, et al., 2020; Shahrour & Dardas, 2020; Xiao et al., 2020). In comparison with previous studies of nurses working in emergency rooms and critical care units pre‐COVID‐19 pandemic, workload and witnessing patient's suffering have been sources of stress among nurses (Brandford et al., 2016; Janda et al., 2015; Al‐Abdallah et al., 2019). In addition, wearing PPEs for long hours caused a lot of physical discomfort among nurses. Some nurses also questioned the quality of the PPEs provided by the hospital. Managing COVID‐19 patients and adhering to infection control procedures is associated with increased workload for nurses, including donning and doffing procedures of PPEs and intensive care workload needed by critically ill COVID‐19, such as intubation, central line and arterial line insertions, changing positions, and so on. (Giuliani et al., 2018). In the study of Lucchini et al. (2020), the nursing workload among those who worked with COVID‐19 patients in ICU increased by 33%, while Bruyneel et al. (2020) showed that the workload increased by 20%, showing more load during the night shift (27% night shift vs. 21% morning shift) (Bruyneel et al., 2020).

Furthermore, nurses must wear complete PPEs (headcover, N95 mask, face shield or goggles, gown and shoe cover) for long hours to keep them safe especially during aerosol‐generating procedures. In the current study, nurses found wearing PPEs for long hours uncomfortable and was associated with reports of sweating, headache, suffocation and injuries to the face. These findings were consistent with the findings of recent research highlighting challenges associated with wearing PPEs faced by health‐care workers managing COVID‐19 patients. For example, wearing protective masks and clothing cases was identified as a main stressor by frontline medical staff (Xiao et al., 2020). Several reports also showed that wearing PPEs was associated with physical discomfort, facial pressure injuries, chest pain, anoxia, headache, visual disturbances and dermatitis (Atay & Cura, 2020; Bruyneel et al., 2020; Liu, Luo, et al., 2020; Ong et al., 2020; Shoja et al., 2020; Singh et al., 2020).

Fear is a negative emotion that foster behaviours that can impact the physical and psychological well‐being of nurses (Espinola et al., 2016; Harper et al., 2020; Yıldırım et al., 2020). Because of the fear of COVID‐19, frontline nurses were motivated to develop defensive behaviours (Espinola et al., 2016). Fear of COVID‐19 prompts nurses to foster behaviours to protect themselves and their families. In Hubei, China more than 3,000 medical staff were infected by the COVID‐19 during its early stages which cause fear among health‐care providers (Liu, Luo, et al., 2020). Infection among nurses during outbreaks has always been a problem; health‐care workers reported persistent fear because of the highly contagious nature of the virus and during the SARS crisis in Canada trust in equipment/infection control initiatives was negatively related to emotional exhaustion and state anger (Liu, Luo, et al., 2020; Marjanovic et al., 2007). Lack of appropriate knowledge and skills in emergency disaster rescue and training related to covid‐19 were also associated with increased fear (Labrague & de Los Santos, 2020; Liu, Zhai, et al., 2020). This can compromise patient care and debilitate the workforce. It is necessary to further investigate the fear of COVID‐19 among nurses in Qatar to establish measures or policies to improve their working conditions to safeguard their physical and mental health.

Nurses play the most crucial role in managing COVID‐19 patients, and therefore, stressors experienced by nurses can negatively impact the quality of patient care (Karimi et al., 2020). Nurses care for patients 24/7 thereby placing them at high risk of being infected. In addition, the therapeutic relationship and the specialized type of care evolved an emotional impact for nurses' caring of prolonged suffering patients in intensive and emergency care units (Alharbi et al., 2019) which most participants of this study clearly stated. One of the unique aspects of the COVID‐19 pandemic is that majority of nurses' witness patients dying alone. Because of hospital policy, family visits were prohibited to prevent further spread of the infection. This led nurses to use their smartphones to connect patients to their loved ones causing ethical dilemmas on privacy and hospital rules (Wakam et al., 2020). A creative approach should be evaluated to allow physical and virtual visits and thereby improving patient's satisfaction and outcomes and nurses' morale (Ganeshan et al., 2021; Rose et al., 2021).

The capacity of nurses to recover from adversities during COVID‐19 was evident in our study. Nurses in Qatar demonstrated self‐care and protective behaviours which can be both beneficial and harmful. Some of the participants in our study made conscious effort to stay fit through exercise and eating right while others reported eating more in response to stress. There was a common knowledge among some of the participants that eating a lot would increase their immunity. Consuming high energy‐dense foods is a typical response to stress; some studies show that stress has a significant correlation with weight gain (Niu et al., 2017; Tsai et al., 2019). During the COVID‐19 pandemic change in eating habits was a common coping mechanism for nurses working in Saudi Arabia (Alhusseini & Alqahtani, 2020). Stress and the change of shifts can influence how and what nurses eat and may contribute to weight gain and obesity (Almajwal, 2016; Karakaş, 2020).

Another interesting finding is that nurses in Qatar were practicing extra safety measures, such as washing uniforms separately, sealing mobile phones with plastic and wearing N95 mask on top of a surgical mask, without existing proof of reducing the risk of infection during the time of this study. Most participants reported stricter compliance to hand washing and other infection control practices compared to pre‐COVID‐19. Nurses who lived with their families deprived themselves of physical interaction with their loved ones which is consistent with previous findings (Kackin et al., 2020; Lee & Lee, 2020). Regular screening of nurses is necessary to assure nurses that they are free from infection and thereby reducing their worries (Lee & Lee, 2020). Infection control practitioners must update nurses on evidence‐based practices in protecting themselves from contagious diseases like COVID‐19. Hospital administrators should install a decontamination area in a COVID‐19 facility where nurses can dispose of used scrubs and wash prior to leaving the hospital. Expanding the provision of temporary living space for nurses with families should also be considered.

In our study, nurses working in a COVID‐19 facility in Qatar revealed that teamwork existed all throughout the pandemic. Despite coming from different facilities where conflicts may arise, nurses in this study found a way to work together during the COVID‐19. Some participants even described the relationship between nurses as a family despite knowing them for less than six months. This global phenomena of camaraderie and working as a team during the COVID‐19 crisis helped nurses in coping with the challenges as one acknowledges the importance of caring for another nurse and sharing the load (Jassar et al., 2020). It is important to note that majority of nurses in Qatar are expatriates. Therefore, it is vital for nursing leaders to recognize and continue fostering this sense of belongingness and team spirit among nurses (Liu, Luo, et al., 2020).

Frontline nurses in a COVID‐19 facility in Qatar showed resilience and willingness to work amidst the risks of getting infected. This was supported by the study of Nashwan, Abujaber, et al., (2020) where nurses in Qatar were willing to take care of patients infected with COVID‐19 (Nashwan et al., 2020). Consistent with other studies, majority of nurses were passionate and were willing to be at the frontlines in the battle against a formidable virus because they find it as a calling and a part of their jobs (Lee & Lee, 2020; Liu, Luo, et al., 2020; Liu, Zhai, et al., 2020; LoGiudice & Bartos, 2021).

The health and safety of frontline nurses should be emphasized during a time of the pandemic. Nurses should feel safe and comfortable when treating patients during a viral pandemic like COVID‐19. There should be an avenue for nurses to safely raise their concerns in the workplace. Maximum working hours and shift arrangements must be favourable to allow more rest periods to prevent and minimize the workload. In addition, mental health support should be easily accessible and assessment be provided for all frontline nurses; and there should be a consistency in the quality and provision of PPEs among nurses (Liu, Luo, et al., 2020; Shoja et al., 2020). Furthermore, there must be a worldwide commitment to improving the comfortability of wearing PPEs. Self‐care must be promoted among nurses and authorities should make every effort to provide a safe venue for recreation. It is at utmost importance that government and health‐care leaders and the population, in general, provide comprehensive support to frontline nurses during the current COVID‐19 pandemic as well as potential future communicable disease outbreaks and pandemics.

## 5 CONCLUSION

Frontline nurses during the COVID‐19 pandemic in Qatar faced many challenges that compromised their physical, emotional and psychological well‐being. However, despite all the adversities, nurses in Qatar were willing to work and take care of patients infected with COVID‐19. Government and health‐care leaders should lead an example in providing comprehensive support to nurses to protect their well‐being. Policies and protocols should be established to mitigate and anticipate stressors brought about by the catastrophic crisis in a pandemic.

## 6 LIMITATIONS

This phenomenological study explored and described the lived experiences of frontline nurses during COVID‐19 in Qatar. The study was limited in scope since we only interviewed 30 participants in a single COVID‐19 facility. Another limitation was the possibility of selection bias and uneven distribution of nurses basing on gender and expertise. Lastly, the interview was done months after the peak of COVID‐19 cases in Qatar and responses might have been different if it was conducted during the initial and peak cases of COVID‐19.

## CONFLICT OF INTEREST

None.

## AUTHOR CONTRIBUTIONS

RCV, AJN, RGM, ASM, SM, AAA, MMA: Research design, Data collection, Analysis, Literature search, Manuscript preparation. MS: Manuscript editing. All authors read and approved the final manuscript.

## Data Availability

All data generated during this study is included in this published article.
